# Mr. West's Address

**Published:** 1911-07-01

**Authors:** A. William West

**Affiliations:** Belgrave Square, S.W.


*To the Editor of* The Hospital.

### MR. WEST'S ADDRESS.

*To the Editor of* The Hospital.

Dear Sir,—I see in The Hospital for June 24 the architect for the new sanatorium proposed at Binton Hill is stated to be Mr. West, of the County Hall, Warwick. This is an error, as my address is as below. As many letters are being sent to me at the County Hall, Warwick, it would be a kindness if you could have this corrected.

3a Lyall Street, Yours truly,

Belgrave Square, S.W. A. William West.

June 26.

